# Hyperspectral Image-Based Night-Time Vehicle Light Detection Using Spectral Normalization and Distance Mapper for Intelligent Headlight Control

**DOI:** 10.3390/s16071058

**Published:** 2016-07-08

**Authors:** Heekang Kim, Soon Kwon, Sungho Kim

**Affiliations:** 1 Department of Electronic Engineering, Yeungnam University, 280, Daehak-ro, Gyeongsan-si, Gyeongsangbuk-do KS011, Korea; kimhk@ynu.ac.kr; 2 Daegu Gyeongbuk Institute of Science and Technology, 333, Techno jungang-daero, Hyeonpung-myeon, Dalseong-gun, Daegu KS002, Korea; soonyk@dgist.ac.kr

**Keywords:** intelligent transportation system, Intelligent Headlight Control, headlight detection, hyperspectral image, rear lamp detection, spectral distance

## Abstract

This paper proposes a vehicle light detection method using a hyperspectral camera instead of a Charge-Coupled Device (CCD) or Complementary metal-Oxide-Semiconductor (CMOS) camera for adaptive car headlamp control. To apply Intelligent Headlight Control (IHC), the vehicle headlights need to be detected. Headlights are comprised from a variety of lighting sources, such as Light Emitting Diodes (LEDs), High-intensity discharge (HID), and halogen lamps. In addition, rear lamps are made of LED and halogen lamp. This paper refers to the recent research in IHC. Some problems exist in the detection of headlights, such as erroneous detection of street lights or sign lights and the reflection plate of ego-car from CCD or CMOS images. To solve these problems, this study uses hyperspectral images because they have hundreds of bands and provide more information than a CCD or CMOS camera. Recent methods to detect headlights used the Spectral Angle Mapper (SAM), Spectral Correlation Mapper (SCM), and Euclidean Distance Mapper (EDM). The experimental results highlight the feasibility of the proposed method in three types of lights (LED, HID, and halogen).

## 1. Introduction

**Figure 1:**
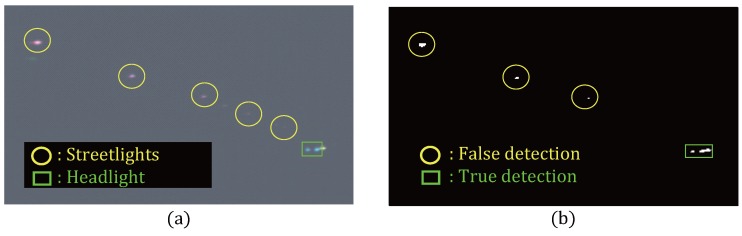
Previous headlight detection method: (**a**) composite image by extracting the RGB bands in the hyperspectral camera (**b**) binary image of headlight detection using the OTSU algorithm [12].

Intelligent headlight control (IHC) is important for advancing the driver assistance system (ADAS) for drivers who drive at night [1]. IHC systems aim to adjust the high beam of a vehicle automatically to illuminate the road ahead. The higher rate of vehicle accidents at night than during the day highlights the need for an IHC system [2]. Another important topic is the improvement of the visibility on the road at night [3]. Some detection techniques for IHC systems have been used, such radar system [4], microwave detectors [5], ultrasonic sensors [6], and infrared thermal sensors. These methods, however, are very expensive and the recent research trends have been in vision-based intelligent night-time driver assistance and surveillance system (VIDASS system) using a dashboard camera, because of its low cost and potential to collect a large amount of information [7]. Two kinds of cameras are available to develop an IHC system: Complementary metal-Oxide-Semiconductor (CMOS) [8] and Charge-Coupled Device (CCD) camera [9]. CMOS camera is image sensor as a type of integrated circuits, CCD camera is image sensor as a device for the movement of electrical charge. Research on vehicle detection during the day has been reported [10], but it is not useful at night. Other vehicle light detections systems have been reported, such as a grayscale image [11] and the OTSU algorithm [12] or blob detection technique [13,14,15]. In addition, methods using these studies used a Region of Interest (ROI) [12,16]. A probabilistic tracking method which can be used to represent the associations of two blobs from different frames, has also been reported [17]. Another detection method using R3I sensors, which have different appearance features and are used as the input for a novel classifier-based module, yields a degree of resemblance to a vehicle light [18]. These studies can be used in hyperspectral image processing, because visible hyperspectral images include the red, green, blue (RGB) bands, if the hyperspectral camera has a high frame rate. Figure 1 shows the limitations of the CCD-based headlight detection using the threshold method; (a) is a composite image from RGB bands in hyperspectral image; and (b) is the result of headlight detection as a binary image. This method, however, is not perfect for headlight detection, as shown in Figure 1, because of the following problems. First, other lights can be detected as targets (headlights), such as signboard lights, streetlights and traffic lights as shown in Figure 1 (in case of street detection as a headlight). Second, some studies used a ROI, but the position of the ROI can change according to the location of the installed camera. Therefore, this study examined another way of detecting vehicle headlights using hyperspectral images. Hyperspectral images are collected at very narrow wavelength intervals [19]. In addition, hyperspectral images provide more information than multispectral images and CCD images. Hyperspectral images combine the features of the images and spectroscopy to acquire both spatial and spectral data simultaneously [20]. For example, Figure 1 presents an image where each pixel has spectral data as shown in Figure 3. In the case of this paper, there is a great deal of spectral information in each pixel because the number of bands is 258. Therefore, there are many applications using hyperspectral image, such as damage detection of fruit spots in the food quality field [21], and distinguishing cancer [22] or tumors [23] in the medical field. This paper conducted experiments to detect vehicle headlights such as light emitting diodes (LED), high-intensity discharge (HID), and halogen, to develop an IHC system. In headlight lens, glass dispersion alters the position of the focal point according to the wavelength. For example, the focal point of blue light is closer to the lens than that of red light because the refraction index of blue light is typically higher than that of red light [24]. This paper proposes a headlight detection method using hyperspectral images instead of CCD or CMOS cameras. In addition, this paper compares each method (Raw data Mean-centered normalization) using detection metrics such as Spectral Angle Mapper (SAM), Spectral Correlation Mapper (SCM) and Euclidean Distance Mapper (EDM). Section 2 explains the purpose of this research and scenario. Section 3 describes the proposed vehicle headlight detection system using the detection metric after processing normalization. Section 4 presents the experiment results. Section 5 reports the conclusions.

## 2. Research Objectives and Experiment Scenario

**Figure 2:**
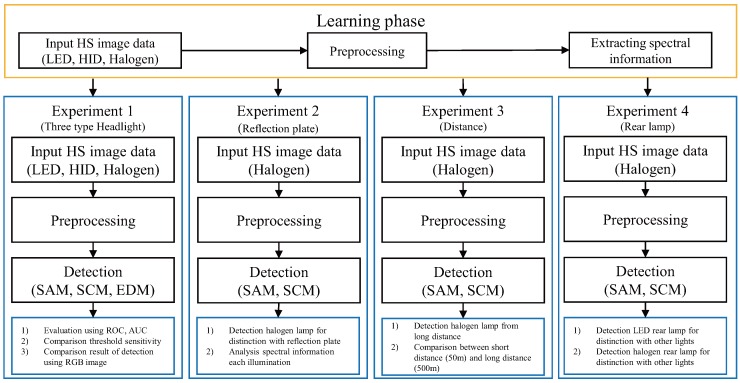
Block diagram of processing to detect vehicle headlights and a comparison of each method.

A hyperspectral camera sensor must be commercialized to apply the IHC technique using the hyperspectral imaging technology. In particular, the lens design is a very important part. The projected area of a lamp can be calculated geometrically from a pixel illumination model. The larger the lens magnification, the larger the projected area of the lamp in an image. If the projected area of the lamp is less than 1, the lamp fits only into a single pixel [25]. This paper shows four types of results in the experiments. The first experiment is headlight detection, such as LED, HID, and halogen lamp distinguish between headlights and street lamps. The second experiment is focused on headlight detection for the distinction from the reflection of the ego-car in the infrastructure. The third experiment is related to the headlight detection capability according to the target distance (maximum 500 m). The final experiment is a rear lamp detection, such as LED and halogen lamp. Headlight detection should be made at a distance of at least 750 m in a straight line. One the other hand, it is very difficult to get a sufficient distance for the experiment. Therefore, this paper compared the headlight detection capability according to a target distance of 50 m and 500 m. This paper shows the research results of spectral analysis for active high-beam control. Therefore, it will assist in studies of IHC using spectral information in the future. A hyperspectral image can be used for lane markings detection and traffic sign recognition by extracting some band (like RGB band), if the hyperspectral camera has a high resolution and frame rate, such as current dashboard camera. The current state-of-the-art hyperspectral sensor can provide 16–25 bands with frame rate of 10 Hz. Figure 2 summarizes the headlight detection flow including a comparison with preprocessing methods, detection methods and an evaluation of the performance of each method and previous method. Three types of headlights were used, LED, HID and halogen. Preprocessing can be divided into two types. First, the RGB data is extracted from a hyperspectral image; its red, green and blue band is 639 nm, 549.41 nm, and 457.97 nm respectively. The Mean-Norm is Mean-centered normalization [26]. Headlights are detected using a spectral normalization and spectral distance mapper, such as Spectral Angle Mapper (SAM), Spectral Correlation Mapper (SCM) and Euclidean Distance Mapper (EDM).

## 3. Proposed Vehicle Headlight Detection System Using Hyperspectral Image

**Figure 3:**
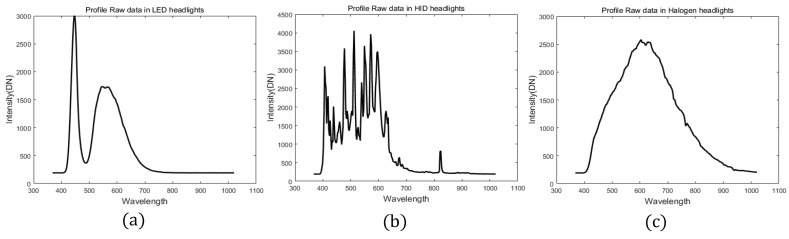
Measured spectral profiles of headlights (**a**) LED, (**b**) HID and (**c**) Halogen.

Figure 3 presents the spectral profile (in 10 random points) when the distance is 300 m from the hyperspectral camera to the vehicle. The peak value in the LED headlight is observed at 445.71 nm in the visible spectrum because LED lamps do not emit ultraviolet or infrared wavelengths. This makes it, more efficient to compare them with other light sources, such as halogen and HID lamps [27]. HID lamps are a source of light that is produced by the radiant energy generated from a gas discharge [28]. Halogen lamps emit light when a tungsten filament is heated to high temperatures [29]. Therefore, each head light source has different features.

**Figure 4:**
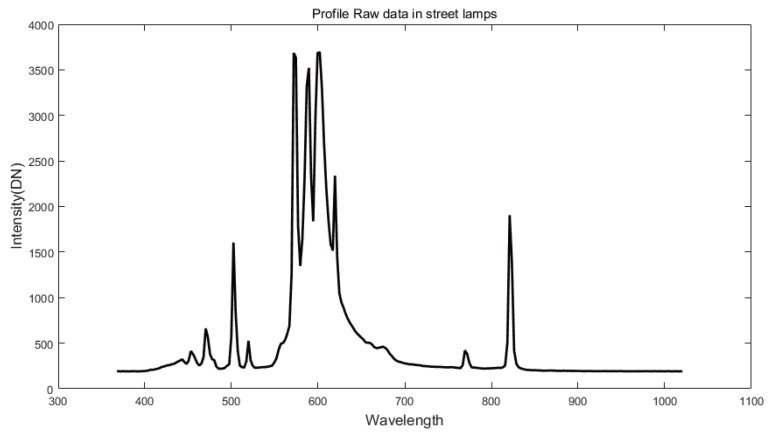
Spectral profile of a streetlight.

Figure 4 presents the spectral profile of a street lamp. The street lamps have different spectral profile information from headlamps.

### 3.1. Preprocessing Spectral Profile (Normalized Spectral Profile)

**Figure 5:**
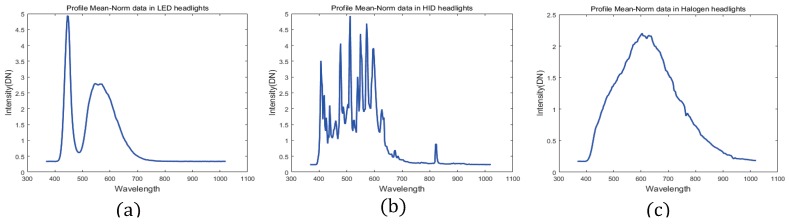
Comparison of the spectral profiles after preprocessing in the Mean-Norm (**a**) LED headlight (**b**) HID headlight (**c**) Halogen headlight.

Normalization techniques are used to solve the problems generated by different spectral profiles from the same material due to shading and shadow effects [26]. A range of normalization methods are used. Max-Norm is a Max-normalization method that divides the spectral profile using the maximum value in a pixel [30]. And Norm-Norm is Norm-normalization, which divides the spectral profile from the norm value in a pixel [31]. The most common technique is the same as the a Mean-Norm which is the same as mean-centered normalization [32] and can be expressed as Equation (1):

(1) $$I\left(P,\lambda \right)=c_{0}\frac{i(P,\lambda )}{\frac{1}{N}\sum_{\lambda =1}^{N}i\left(P,\lambda \right)}$$

where I(P,λ) is a normalized spectral profile intensity in the $P_{th}$ pixel at the $\lambda_{th}$ band in a hyperspectral image, and i(P,λ) is the raw spectral profile in a hyperspectral image. N is the total number of bands (In case of this paper, the number of bands is 258), $c_{0}$ is the scale factor to make an appropriate value to see an image [30]. However, it is possible to omit the constant $c_{0}$. In Equation (1), this intensity value I(P,λ) is divided by the mean of the raw spectral profile. Figure 5 presents three headlights after processing the Mean-Norm; (a) is the LED headlight profile (b) is the HID headlight and (c) is the halogen headlight.

### 3.2. Processing Headlight Detection

This study used three detection metrics, SAM, SCM, and EDM. These methods were compared in each headlight type (LED, HID and Halogen).

#### 3.2.1. Spectral Angle Mapper (SAM)

The SAM algorithm is based on the ideal assumption that a single pixel of remotely sensed images represents a certain ground cover material, and can be assigned uniquely to only one ground cover class. The SAM algorithm is just a simple measurement of the spectral similarity between two spectra [33]. The SAM compares the angle between the spectrum vector and each pixel vector in n-dimensional space in Equation (2), where SAM(P) is the spectral angle between the reference spectra and a test spectra of the $P_{th}$ pixel. The reference spectra is a learned profile and the test spectra is the profile of a pixel in the image to determine if it is a headlamp.

(2) $$SAM\left(P\right)=\cos^{-1}\left(\frac{\sum_{n=1}^{N}i_{ref}\times i_{test}}{\sqrt{\sum_{n=1}^{N}i_{ref}^{2}}\times \sqrt{\sum_{n=1}^{N}i_{test}^{2}}}\right)$$

**Figure 6:**
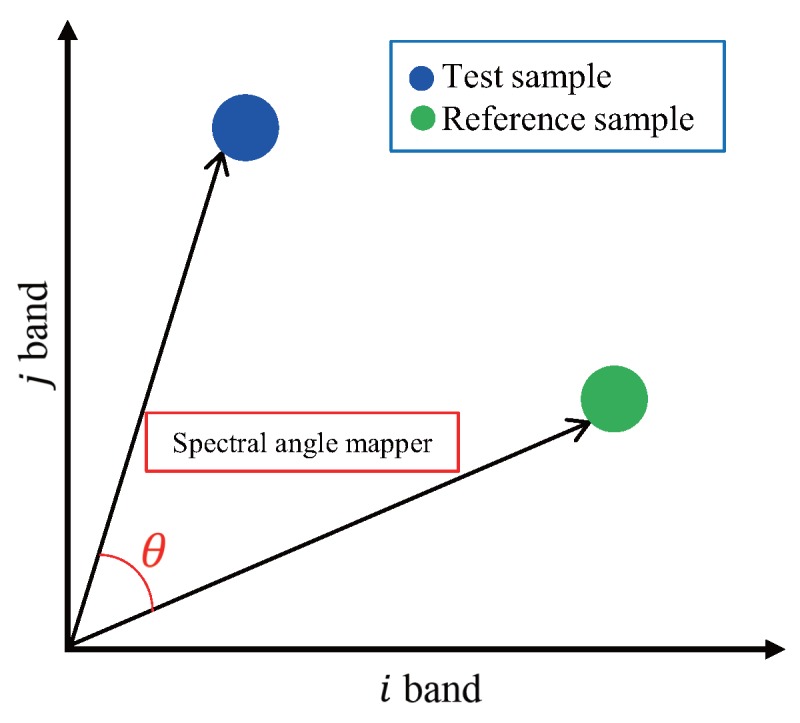
Description of the SAM in the three bands reference [34].

Geometrically, at a lower angle (close to 0${}^{\circ }$), each spectrum has similar materials and the higher the angle (close to 90${}^{\circ }$), each spectra does not have similar materials, as shown in Figure 6.

#### 3.2.2. Spectral Correlation Mapper (SCM)

The SCM was introduced [35] to measure the spectral similarity of the two reference spectral intensity ($I_{ref}$) and test spectral intensity ($I_{test}$).

(3) $$SCM\left(P\right)=\frac{\sum_{n=1}^{N}i_{ref}\times i_{test}-\sum_{n=1}^{N}i_{ref}\sum_{n=1}^{N}i_{test}}{\sqrt{[\sum_{n=1}^{N}i_{ref}^{2}-\sum_{n=1}^{N}{\left(i_{ref}\right)}^{2}][\sum_{n=1}^{N}i_{test}^{2}-\sum_{n=1}^{N}{\left(i_{test}\right)}^{2}]}}$$

where $I_{ref}$ is the average spectrum in each headlight (distance is 300 m from the hyperspectral camera to the vehicle). $I_{test}$ is the difference spectral in the test image (distance is 450 m from the hyperspectral camera to the vehicle is 450 m). The SCM has the advantage in that it has information that is expressed a number.

#### 3.2.3. Euclidean Distance Mapper (EDM)

The EDM is one of the popular spectral similarity measures and has been used widely in multispectral and hyperspectral image analysis. The EDM is expressed as Equation (4) in n-dimensional space [36].

(4) $$EDM\left(P\right)=\sqrt{\sum_{n=1}^{N}{\left(I_{ref}-I_{test}\right)}^{2}}$$

**Figure 7:**
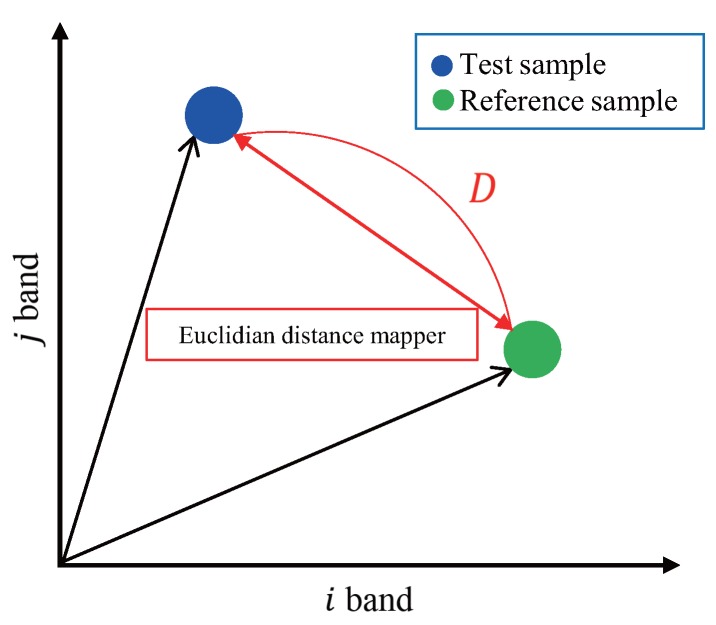
Description of the EDM in two bands.

The Euclidean distance can measure the spectral distance between two spectral profiles in n-dimensional (the number of bands) spectral feature space. Figure 7 presents a description of the EDM in two bands. When the goal of optimization is to minimize the squared error, it can be used by the negative Euclidean distance [37].

## 4. Experimental Results

### 4.1. Hyperspectral Image Acquisition System

The hyperspectral image acquisition system consisted of a SPECIM VNIR (Middleton Spectral Vision, Tallahassee, FL, USA) camera mounted on a rotary tripod (Table 1). The original image contained a total 1032 bands with the highest spectral resolution; however, this study used only 258 bands to detect the headlamp to reduce the number of dimensions. The spectral range was 400–1000 nm. The radiance data was saved in 12-bit binary files. Although the image size was 1392 × 1040 pixels, only half of the data of the total image size, such as 200 × 350 pixels, was used to reduce the data.

**Figure 8:**
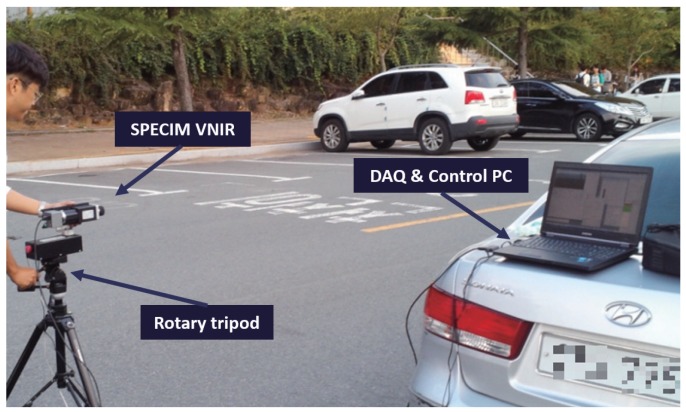
Visible near infrared (VNIR) hyperspectral image acquisition system.

Headlamps were prepared such as Mercedes-Benz′s E-class (LED lamp) (Friedberg, Germany), Hyundai′s Grandeur-HG (HID lamp) (Deagu, Korea), Kia′s Sorento (Halogen lamp) (Deagu, Korea), Hyundai′s Sonata(Halogen lamp) (Deagu, Korea) and Kia′s Cerato(Halogen lamp) (Deagu, Korea). Unlike the detection of reflecting light from a material, this experiment minimized the aperture size of the hyperspectral camera, to detect the light directly from the lamp. Matlab R2015b (The MathWorks Inc., Natick, MA, USA) was used to analyze the spectral profiles and process the headlamp detection. Figure 8 presents the installed visible near infrared (VNIR) hyperspectral image acquisition system on a parking lot in Yeungnam University (Gyeongsan-si, Korea). The distance between the camera and automotive headlamp was 450 m (test image).

### 4.2. First Experiment for Three Type Lamps Detection

Three types of evaluations were used for each method combination for the detection of each headlight (LED, HID and halogen). This experiment can distinguish between each lamp and streetlamps. In the experiments, preprocessing and detection methods were used.

#### 4.2.1. Evaluation Performance Using ROC and AUC in the First Experiment

**Figure 9:**
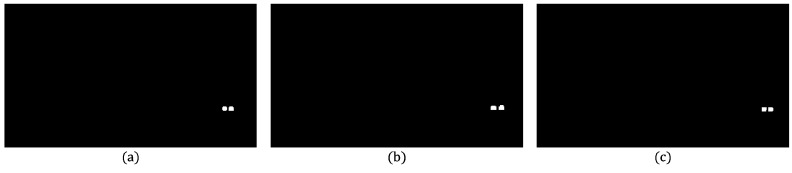
Mask image to calculate the ROC and AUC.

First, a range of methods were evaluated using the Receiver Operating Curve (ROC) and Area Under Curve (AUC); therefore, the best combination can be found by comparing with the previous method to detect a headlight (using OTSU algorithm after converting to grayscale). In the experiment, the detection result can be compared with the AUC in the ROC analysis of detectors. A mask image was made, as shown in Figure 9, to compare the detection methods using the ROC and AUC.

**Figure 10:**
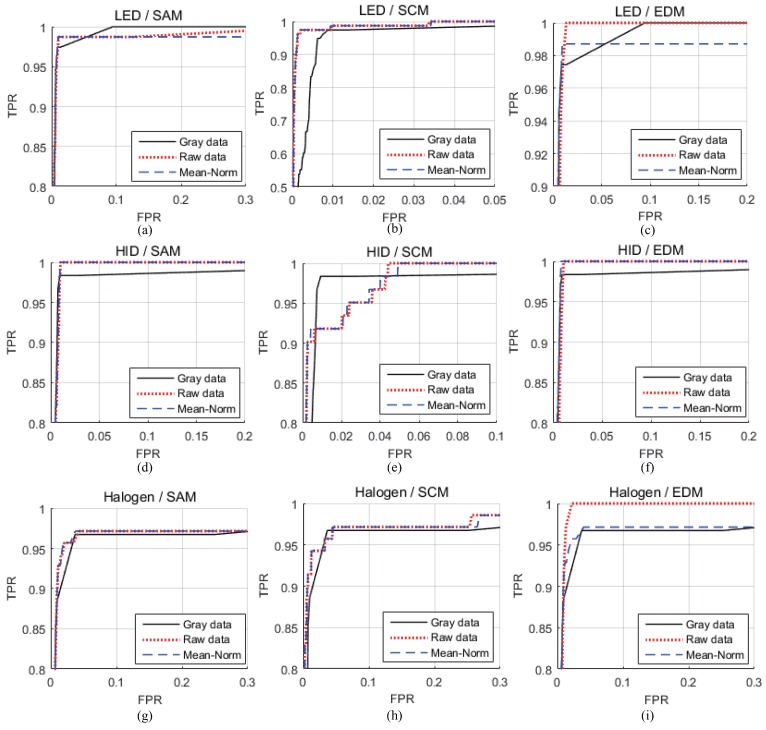
ROC in each combination methods (**a**) using the SAM detection metric in a LED headlight (**b**) using the SCM detection metric in a LED headlight (**c**) using the EDM detection metric in a LED headlight (**d**) using the SAM detection metric in a HID headlight (**e**) using the SCM detection metric in a HID headlight (**f**) using the EDM detection metric in a HID headlight (**g**) using the SAM detection metric in a halogen headlight (**h**) using the SCM detection metric in a halogen headlight (**i**) using the EDM detection metric in a halogen headlight.

Figure 10 shows the ROC result of the detection headlight using each method. The types of headlights are listed at the beginning of each row in the figure and three detection metrics are at the head of in each column figure. Each figure presents, two results from the hyperspectral image (proposed method) and RGB image (previous method). Figure 10 presents the different false positive rate (FPR) value, which is the so-called specificity at the same true positive rate (TPR) value so-called sensitivity in each method [38]. The number of thresholds is 1000 with the same interval.

Table 2 summarizes the AUC metric in each headlight along with a combination with the preprocessing and detection metric.

In the case of the LED headlight, the best detection metric was the SCM and the AUC values were higher than with previous methods using the SCM (Raw, Mean-Norm) and the EDM (Raw). In the case of the HID headlight, the best combination method was the SAM detection metric and Mean-Norm preprocessing. In addition, all AUC values were higher than with previous methods. In the case of the halogen headlight, the best combination method was the EDM detection metric and raw data.

#### 4.2.2. Detection Performance Given the Same False Positive Rate (FPR)

**Figure 11:**
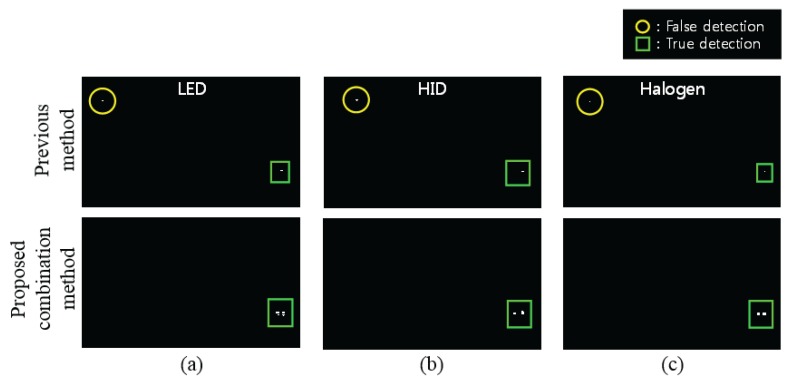
Comparison of the previous method [12] and with proposed method in (**a**) LED, (**b**) HID, (**c**) Halogen.

Figure 11 presents the results at the same FPR and compares the data from the proposed method with that obtained from the previous method [12]. Previous methods detected headlights using grayscale, ROI and blobs in a CCD or CMOS dashboard camera. The proposed method detects headlights using the best combination method in a hyperspectral image. Therefore, the results are compared with grayscale and the best combination method. The detection performance was compared when the FPR was lower than 0.0001. Comparing each detection performance is more effective because the number of target pixels is very low compared to the entire image. In Figure 11, there are three types of headlights, LED, HID and halogen. In a hyperspectral image, the best combinations were the Mean-Norm and SCM in LED, Mean-Norm and SAM in HID and raw data and EDM in Halogen. In the previous method, the thresholds for the LED, HID, and halogen were $337_{th}$, $510_{th}$, and $310_{th}$ in the LED, HID and halogen in the RGB image. In the proposed method, the threshold was $45_{th}$, $565_{th}$ and $421_{th}$ in the hyperspectral image. The total number of thresholds was one thousand.

### 4.3. Second Experiment: Distinction of Headlights from the Reflections of Ego-Car

**Figure 12:**
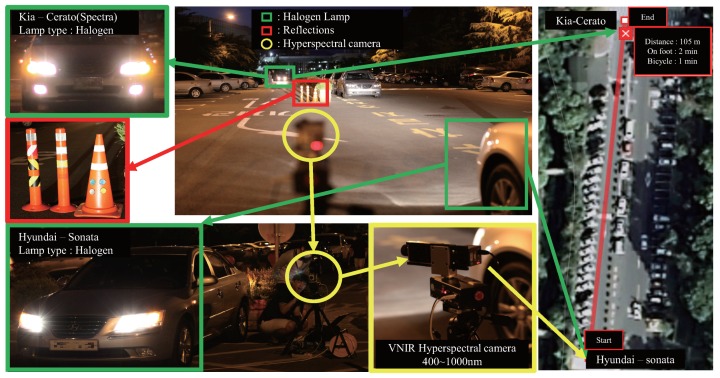
Introduction to the experimental environment.

The second experiment was conducted to distinguish headlights from the reflection of ego-car in the infrastructure. Figure 12 shows the experimental environments. In the center of Figure 12, the green squares represent the halogen headlamps of an incoming car; the red square indicates the reflection posts; the yellow circle indicates the hyperspectral camera.

#### 4.3.1. Comparison of the Spectral Information between Halogen Lamp and the Reflection from a Halogen Lamp

**Figure 13:**
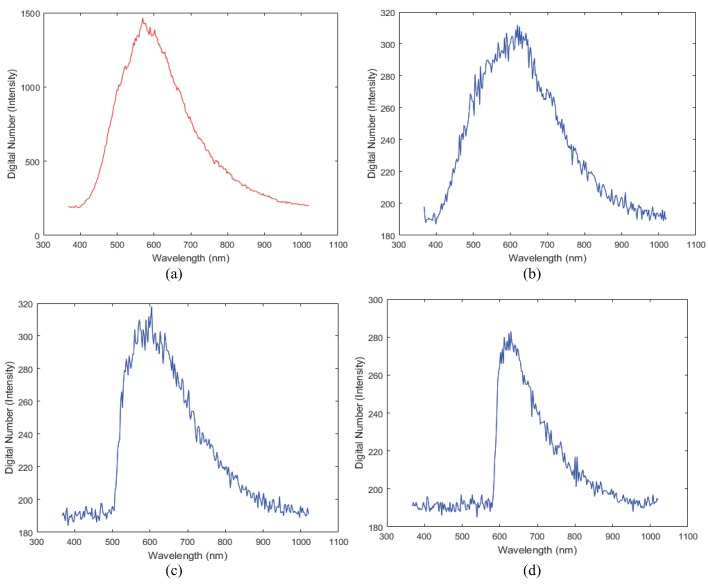
Spectral information of a halogen lamp and reflection: (**a**) halogen lamp spectra (**b**) white reflection spectra, (**c**) yellow reflection spectra, and (**d**) red reflection spectra.

**Figure 14:**
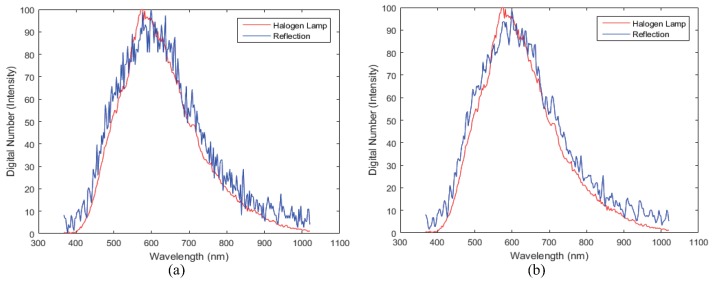
Comparison by the normalized spectral halogen lamp and reflection plate (**a**) without smoothing filter (**b**) with smoothing filter.

The halogen lamp and the reflection of the ego-car show different spectral information. In particular, the yellow reflection, whose spectra is shown in Figure 13c, absorbs in the blue region (380–500 nm). Similarly, the red reflection, whose spectra is shown in Figure 13d, absorbs in the blue and green region (380–580 nm). In addition, the white reflection showed a different spectral profile compared to the halogen lamp, as shown in Figure 14.

#### 4.3.2. Headlight Detection Results in the Reflections of Ego-Car Environment

**Figure 15:**
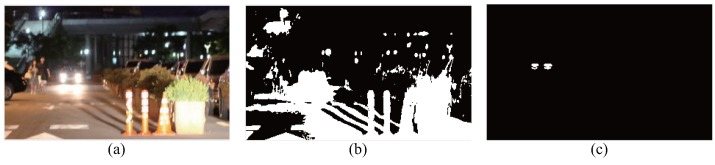
Results of headlight detection (**a**) test scene (RGB picture), (**b**) using the RGB image (**c**) using hyperspectral image.

Figure 15 shows the experimental results of distinguishing the headlights from the reflection plates. Note that the proposed hyperspectral image analysis method can remove the false detections caused by the reflection plates.

### 4.4. Third Experiment to Compare the Headlight Detection from a Near and Far Distance

**Figure 16:**
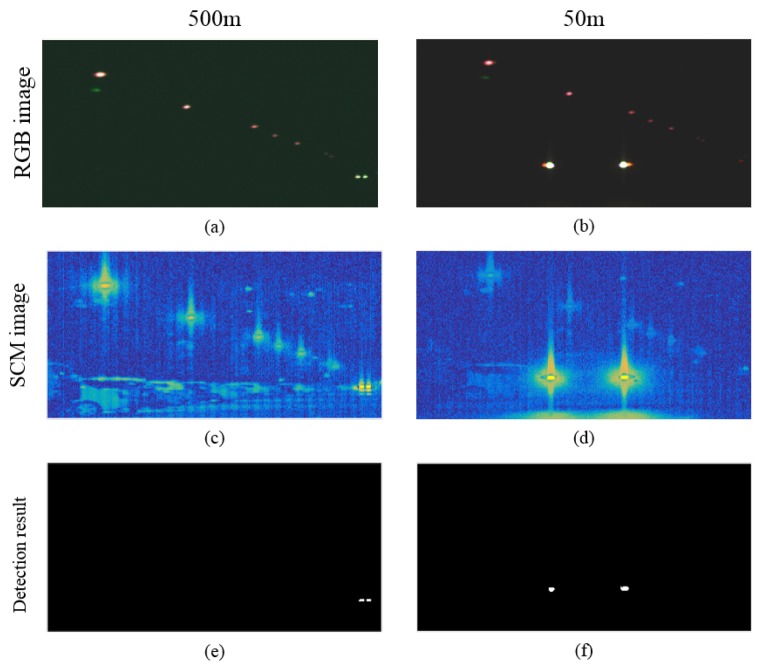
Comparison of the detection results according to the headlight distance: (**a**) RGB composite image acquired at 500 m; (**b**) RGB composite image acquired at 50 m; (**c**) SCM result for the hyperspectral image at 500 m; (**d**) SCM result for the hyperspectral image at 50 m; (**e**) detection result by thresholding to the SAM image for the 500 m data; (**f**) detection result by thresholding to SAM image for the 50 m data.

This experiment compares the detection headlight from the near distance and far distance. Figure 16 compares the headlight detection from the far distance (500 m) and near distance (50 m). The maximum line-of-sight (LOS) distance in authors’ university was 500 m.

The proposed hyperspectral image-based spectral analysis method can detect near and far distant headlights, simultaneously. Almost no degradation of the detection performance depending on the headlight distance was observed because the hyperspectral lamp signatures show consistent spectral profiles.

### 4.5. Fourth Experiment to Detect the Rear Lamps for the Distinction with Other Lamps

Rear lamps are usually in red color, and there are two types of lamps, such as Halogen and LED. The detection of rear lamps is very important in the development of an IHC system.

#### 4.5.1. Comparison Spectral Information Rear Lamps (Halogen and LED)

**Figure 17:**
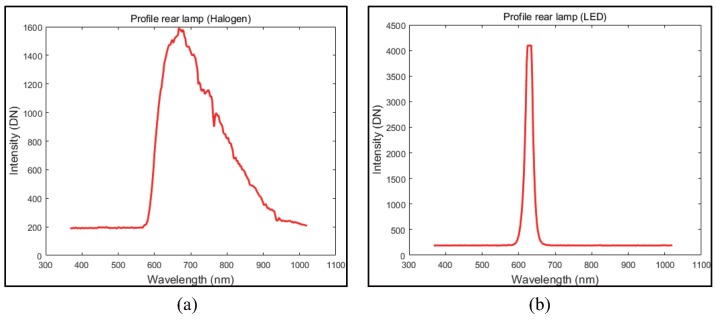
Spectral information rear lamp: (**a**) Halogen rear lamp (**b**) LED rear lamp.

Figure 17 shows the spectral profiles of each rear lamp, such as Halogen and LED. The intensity of the halogen rear lamp is distributed from 650 nm to 900 nm. The intensity of the LED rear lamp has a peak value at 630 nm.

#### 4.5.2. Rear Lamp Detection Result with Other Lights

**Figure 18:**
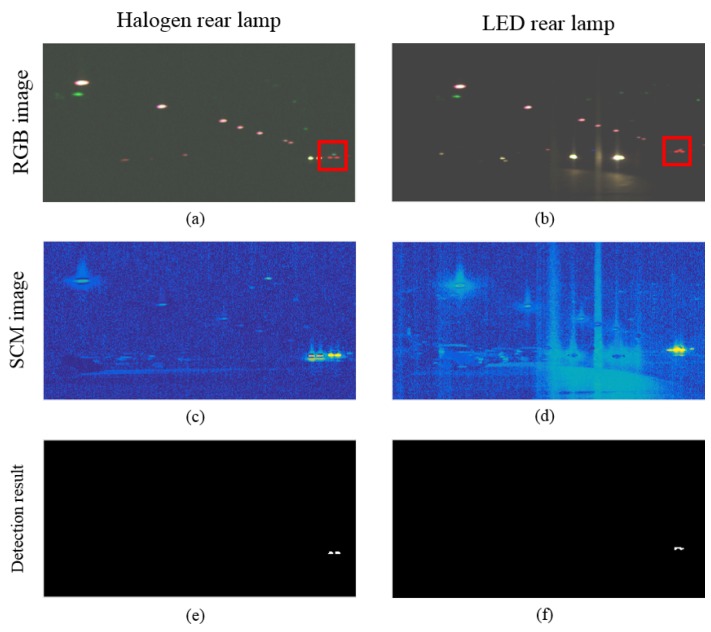
Comparison of the detection of each rear lamp, such as halogen and LED: (**a**) RGB composite image acquired from a halogen lamp; (**b**) RGB composite image acquired from a LED lamp; (**c**) SCM result for the hyperspectral image from a halogen lamp; (**d**) SCM result for the hyperspectral image from a LED lamp; (**e**) detection result by thresholding to SAM image from a halogen lamp; (**f**) detection result by thresholding to SAM image from a LED lamp.

The rear lamp also can distinguish other lights, such as street lamps or other headlights. Figure 17 shows rear lamp detection result at 500 m. In the case rear lamp detection, it is trained as just a rear lamp; other headlights are not detected result in Figure 18e,f.

The proposed hyperspectral image-based spectral analysis method also can detect a rear lamp.

## 5. Conclusions

This paper proposed a new detection method using a hyperspectral image to detect automotive headlights effective for IHC. The experiments were conducted using the SAM, SCM, and EDM distance metrics and the detection performance was assessed using the ROC and AUC methods on real headlight images. In addition, there were two additional experiments. The threshold sensitivity from the previous methods was compared with that of the proposed method. According to the results, the best combination is the SCM detection metric with the raw or Mean-Norm in the case of LED. The best combination is the SAM detection metric with Mean-Norm in the case of HID. The best combination in the case of the halogen lamp was the EDM metric with raw data. The detection performance of the proposed method was lower at the same FPR (≤0.0001). Furthermore, headlights could be detected using a hyperspectral image with a reflection plate. The rear lamp could also be detected with other lights. Nevertheless, future studies will be needed to improve the detection performance using a band selection method to reduce the processing time.

## Figures and Tables

**Table 1:** Specifications of the hyperspectral image acquisition system.

Item	Specifications
Spectral range	400–1000 nm (VNIR)
Spectrograph	ImSpector V10E 30 *μ* slit, 2.8 nm spectral resolution
Camera	Kappa 1392 × 1040 pixels, 12 bits, 11 fps, Firewire interface
Scanner	Rotational tripod, scan angle: max 160${}^{\circ }$

**Table 2:** AUC values in each headlight image (including combinations in each method).

Headlight Type	Detection	Raw	Mean-Norm
LED	SAM	0.9949	0.9857
(OTSU = 0.9964)	SCM	**0.9992**	**0.9992**
	EDM	0.9970	0.9858
HID	SAM	0.9980	**0.9981**
(OTSU = 0.9937)	SCM	0.9965	0.9965
	EDM	0.9970	0.9980
Halogen	SAM	0.9366	0.9351
(OTSU = 0.9800)	SCM	0.9656	0.9654
	EDM	**0.9936**	0.9334
